# Advances in Drug Delivery Systems for Boswellic Acids from *Boswellia serrata*: Enhancing Oral Bioavailability and Therapeutic Efficacy

**DOI:** 10.3390/ijms27104420

**Published:** 2026-05-15

**Authors:** Magdalena Rutkowska, Monika A. Olszewska

**Affiliations:** Department of Pharmacognosy, Faculty of Pharmacy, Medical University of Lodz, 1 Muszynskiego St., 90-151 Lodz, Poland; monika.olszewska@umed.lodz.pl

**Keywords:** boswellic acids, *Boswellia serrata* oleo–gum resin, drug delivery systems, novel formulations, oral absorption, tissues availability, inflammation, biological effects, advantages and limitations

## Abstract

Boswellic acids (BAs), the major bioactive constituents of *Boswellia serrata* oleo–gum resin, exhibit well-documented anti-inflammatory and antioxidant activities, which correspond to their healing effects in arthritis, inflammatory bowel disease, asthma, metabolic syndrome, liver disorders, and certain cancers. However, their therapeutic potential is hindered by their poor aqueous solubility, low intestinal absorption, extensive metabolism, and overall low oral bioavailability. This review provides a comprehensive analysis of conventional *Boswellia serrata* products and advanced drug delivery systems designed to enhance the biological performance of BAs. We summarize recent developments in formulation strategies, including phytosomes, micelles, self-emulsifying drug delivery systems, solid lipid particles, polymeric nanoparticles, hydrogels, cyclodextrin complexes, metal-based nanocarriers, and hybrid delivery platforms. Available in vivo and cellular studies are critically evaluated, with a focus on disease-specific outcomes. Results indicate that emerging formulation technologies significantly increase the oral absorption, systemic exposure, and biological effectiveness of BAs. However, despite promising preclinical data, challenges remain regarding the standardization of *Boswellia* extracts, the stability of novel formulations, their safety, and limited clinical evaluation. By comparing the advantages and limitations of conventional preparations with modern drug delivery systems, this review outlines the most effective strategies to enhance the bioavailability of BAs and highlights future research directions for their translational development.

## 1. Introduction

**Figure 1:**
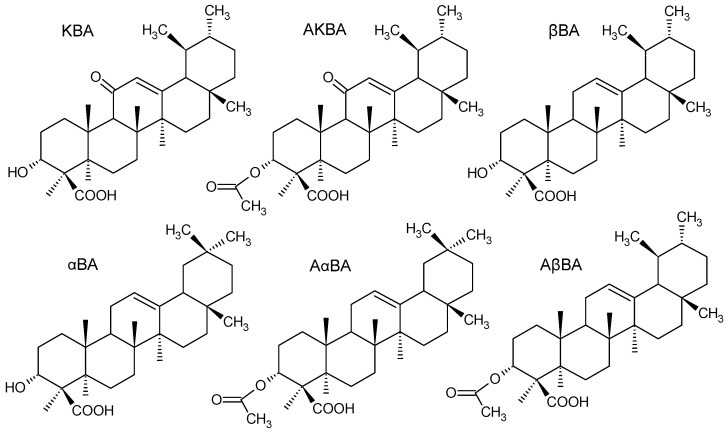
Structures of six primary boswellic acids (BAs) in *Olibanum indicum*: 11-keto-β-boswellic acid (KBA), acetyl-11-keto-β-boswellic acid (AKBA), β-boswellic acid (βBA), acetyl-β-boswellic acid (AβBA), α-boswellic acid (αBA), and acetyl-α-boswellic acid (AαBA).

*Boswellia serrata* Roxb. (family Burseraceae) is a plant used in traditional Ayurvedic medicine, cultivated for its oleo–gum resin, known as *Olibanum indicum*, Indian frankincense, or Kunduru. Frankincense, traded internationally for thousands of years, is currently utilized across various industries, primarily in the pharmaceutical sector, but also in cosmetics and perfumery [1]. It is a complex matrix composed of approximately 5–10% essential oil, 25–30% gum, and 30–60% resin. The volatile oil is a blend of mono-, di-, and sesquiterpenes. The gum is composed of mono-, oligo-, and polysaccharides, and the resin contains monoterpenes, diterpenes, and both tetracyclic and pentacyclic triterpenes [2,3]. So far, more than 30 different triterpenes have been reported in Indian frankincense [4]. Among these, six pentacyclic boswellic acids (BAs, Figure 1) dominate and are primarily responsible for the beneficial health effects of *Boswellia* products. These include 11-keto-β-boswellic acid (KBA, which accounts for approximately 2.5–7.5% of the dry weight (dw) of the oleo–gum resin), acetyl-11-keto-β-boswellic acid (AKBA, 0.1–3% dw), α- and β-boswellic acids (αBA and βBA, 10–21% dw), and acetyl-β- and acetyl-α-boswellic acids (AβBA and AαBA, 0.05–6% dw) [5]. Among the *Olibanum* triterpenes, AKBA has the most significant biological potential [3,5,6,7,8,9,10,11,12,13,14].

**Figure 2:**
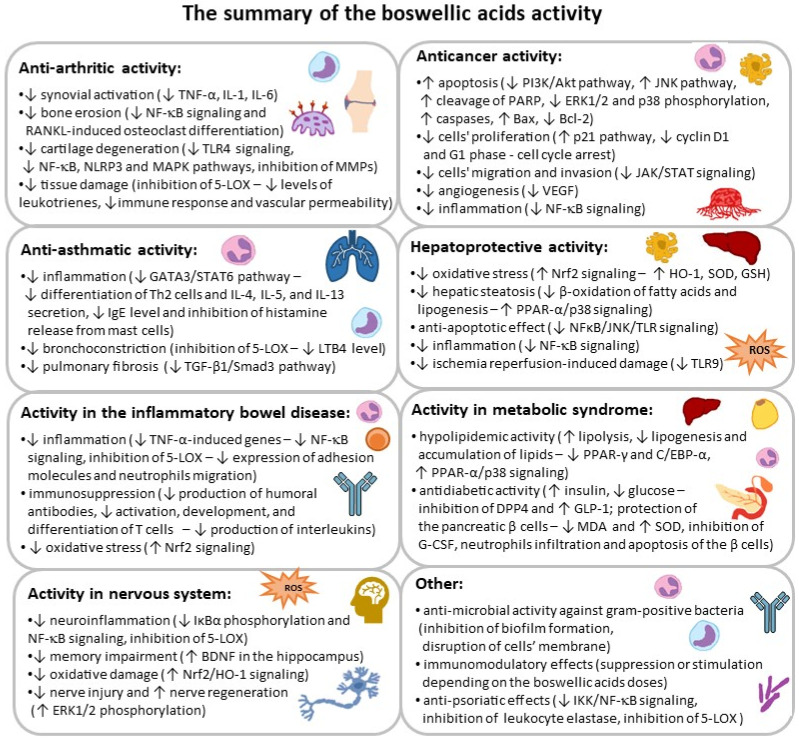
The summary of BAs activity [3,5,6,7,8,9,10,11,12,13,14]. Abbreviations: 5-LOX, 5-lipoxygenase; Akt, protein kinase B; Bax, Bcl-2-associated X protein; Bcl-2, B-cell lymphoma 2; BDNF, brain-derived neurotrophic factor; C/EBP-α, CCAAT/enhancer-binding protein alpha; DPP4, dipeptidyl peptidase 4; ERK1/2, extracellular signal-regulated kinase 1 and 2; GATA3, GATA-binding protein 3; G-CSF, granulocyte colony-stimulating factor; GLP-1, glucagon-like peptide-1; GSH, glutathione; HO-1, heme oxygenase-1; IgE, immunoglobulin E; IKK, inhibitor of kappa B kinase; IL-1, IL-4, IL-5, IL-6, IL-13, interleukins 1, 4, 5, 6, 13; IκBα, inhibitor of kappa-light-chain-enhancer of activated B cells alpha; JAK, Janus kinase; JNK, c-Jun N-terminal kinase; LTB4, leukotriene B4; MAPK, mitogen-activated protein kinase; MDA, malondialdehyde; MMPs, matrix metalloproteinases; NF-κB, nuclear factor kappa-light-chain-enhancer of activated B cells; NLRP3, nucleotide-binding domain, leucine-rich-containing family, pyrin domain-containing-3; Nrf2, nuclear factor erythroid 2–related factor 2; p21, cyclin-dependent kinase inhibitor 1; p38, p38 mitogen-activated protein kinase; PARP, poly(ADP-ribose) polymerase; PI3K, phosphatidylinositol 3-kinase; RANKL, receptor activator of nuclear factor kappa-B ligand; Smad3, small mother against decapentaplegic homolog 3; SOD, superoxide dismutase; STAT, signal transducer and activator of transcription; TGF-β1, transforming growth factor-beta 1; TLR4, toll-like receptor 4; TNF-α, tumor necrosis factor alpha; VEGF, vascular endothelial growth factor; ↓, decrease; ↑, increase. All icons were prepared by the author with the use of PowerPoint and Paint programs.

BAs and AKBA have been reported to possess potent, multifaceted biological activity (Figure 2), particularly anti-inflammatory and antioxidant properties, which correspond to their healing effects in a variety of conditions, including arthritis, asthma, inflammatory bowel disease, metabolic syndrome, liver disorders, and certain cancers. BAs effectively affect various pro-inflammatory signaling pathways associated with the development and progression of these disorders, e.g., MAPKs (mitogen-activated protein kinases), PI3K/Akt (phosphatidylinositol 3-kinase/protein kinase B), TLR4 (toll-like receptor 4), NF-κB (nuclear factor kappa-light-chain-enhancer of activated B cells), JAK/STAT (Janus kinase/signal transducer and activator of transcription), Nrf2 (nuclear factor erythroid 2–related factor 2), and 5-LOX (5-lipoxygenase) [3,6,7,8,9,10,11,12,13,14]. Due to these effects, there is a significant commercial demand for *Olibanum* extracts, with the global market for frankincense reaching thousands of metric tons and hundreds of millions of USD annually [1]. The resin or *B. serrata* extract (BE) also has its monographs in numerous pharmacopeias, including the European Pharmacopeia (Ph. Eur. 11.3, 2023), The United States Pharmacopeia (USP 43, 2010), and The Ayurvedic Pharmacopeia of India (2004) [15,16,17].

Despite this extensive interest, the clinical application of *Boswellia* preparations remains relatively limited. A primary barrier is the inherently poor oral bioavailability of BAs, especially AKBA, due to their high lipophilicity, low aqueous solubility, intestinal instability, and extensive metabolism. The bioavailability of AKBA in humans following oral administration of crude *Boswellia* extracts has been estimated at 0.24–0.35% under fasting conditions and up to 1.66% with a high-fat meal [18,19,20]. Other BAs demonstrate slightly improved but still suboptimal absorption, with wide inter-study variability (6.63–8.95% for KBA, 2.69–25.58% for βBA and αBA, and 2.99–39.62% for AβBA and AαBA).

Efforts to enhance the efficacy of BAs using conventional phytochemical approaches have included enriching extracts with AKBA by the semi-synthetic conversion of βBA/αBA to AKBA [21]. Although these strategies increase the AKBA concentration in extracts (from a typical 1–10% to as high as 100% dw), they still fail to overcome the low absorption and restricted tissue distribution of BAs. Even formulations combining extracts with some solubility enhancers, such as the oil-enriched Aflapin^®^, while achieving improved serum BAs levels in animals, showed only modest improvements in a clinical trial of osteoarthritis compared with the conventional extract [22]. The observed disconnect between increased plasma exposure and limited tissue distribution underscores the need for advanced formulation strategies.

Consequently, recent research has shifted towards developing novel drug delivery systems that enhance solubility, protect against degradation, improve membrane permeability, and enable targeted or controlled release of BAs. Over the past decade, various lipid-, polymer-, inorganic-, and hybrid-based delivery platforms have been developed to enhance the pharmacokinetic and pharmacodynamic performance of both pure BAs and *Boswellia* extracts.

Existing review articles on *Boswellia serrata* provide valuable insights into its pharmacological properties, molecular mechanisms of action, and phytochemical characteristics [3,5,6,8,9,12,14,23,24,25,26,27,28,29,30]. Additionally, *Boswellia* is frequently discussed in broader disease-oriented reviews focused on natural products or complementary therapeutic strategies, where it appears alongside other bioactive compounds (e.g., curcumin) as one of several supportive agents rather than as the primary subject of analysis (e.g., [31,32,33,34,35]). Some previous reviews also mention formulation strategies for *Boswellia*, but focus on selected formulation examples or general overviews of formulation approaches. Some of them included topical formulations, synergistic multi-component preparations, simple formulation approaches aimed primarily at solubility enhancement rather than engineered drug delivery systems, formulations involving *Boswellia* species other than *Boswellia serrata*, or studies lacking comparison with non-formulated extracts, which limited the assessment of formulation-dependent improvements in bioavailability or biological activity [36,37,38,39,40,41,42,43,44,45,46,47,48].

Therefore, although the existing literature is scientifically valuable within its respective scopes, it does not provide a comprehensive and focused analysis on engineered oral drug delivery systems specifically developed for *Boswellia* serrata-derived triterpene acids. This observation, supported by an initial literature review, motivated the selection of the present topic and highlighted the need for a dedicated, structured analysis of *Boswellia*-based formulation strategies.

This review analyzes twenty-seven modern delivery systems developed over the last fifteen years, comparing their technological characteristics, pharmacokinetic advantages, and biological performance with those of the corresponding non-formulated extracts. Additionally, key challenges, limitations, and future perspectives for optimizing BAs delivery and translating next-generation formulations into clinically relevant therapeutic options have been outlined.

## 2. Methods—Literature Search Strategy

### 2.1. Gap Identification and Topic Selection

Prior to the formal literature search, an initial screening of existing review articles was conducted (Scopus, Web of Science, Embase, ScienceDirect, PubMed, Google Scholar) to evaluate the current state of knowledge and identify gaps in the literature. The first step involved a targeted search using general keywords such as “*Boswellia*”, “boswellic acid”, and “frankincense”. Following this, a more focused search was conducted that combined specific terms like “*Boswellia serratta*”, “delivery systems”, “nanocarriers”, and “particles”. Although both search steps yielded numerous records, a detailed analysis revealed that none of the retrieved reviews thoroughly addressed the specific scope of the current study. Specifically, manual screening and a cross-analysis of reference lists showed that individual formulation-oriented reviews only covered a limited subset of the available primary literature on the topic. Typically, they included 1–8 of the 30 original papers incorporated into the current review (see Section 2.2). Consequently, the preliminary assessment confirmed the need for a focused synthesis of original research on *Boswellia*-based drug delivery systems.

### 2.2. Main Literature Search

The main literature search focused on identifying original research articles on *Boswellia* extract (BE) and BAs formulations. A structured search strategy was applied across multiple electronic databases (Scopus, Web of Science, Embase, ScienceDirect, PubMed, Google Scholar), targeting articles published between January 2000 and December 2025 (however, it turned out that studies on *Boswellia* formulations started in 2010).

To maximize sensitivity, the initial search employed broad, general keywords (i.e., “*Boswellia serrata*” and “boswellic acid”). In the context of emerging drug delivery systems, exclusive reliance on controlled vocabulary (e.g., MeSH terms) at the outset may be insufficient, given the inconsistent indexing and heterogeneous terminology used to describe novel delivery systems. A repeated search process was therefore implemented. Following preliminary screening, additional, more specific keywords related to formulation types and delivery systems (“micellar formulations”, “phytosomes”, “self-emulsifying drug delivery systems”, “solid lipid particles”, “nanospheres”, “nanocapsules”, “hydrogels”, “cyclodextrins inclusion complexes”, “solid dispersions”, “metal-based nanoparticles”, “layered double hydroxides”, “hybrid drug delivery systems”, “polymer-lipid drug delivery systems”, and “polymer-inorganic drug delivery systems”) were incorporated and combined using Boolean operators (AND/OR) to sharpen the search results. Reference lists of relevant publications were also screened (hand-searching) to identify additional eligible studies.

The duplicates were removed, and the records were validated by reading the title, abstract, and/or the entire paper to ensure they addressed BE or BAs formulations, excluding studies focused solely on raw *Olibanum* or non-formulated extracts. The plant name was checked and revised in accordance with the World Flora Online Plant List [49].

Only original research articles were included in the main analysis and discussion. Review articles were intentionally excluded from this stage to ensure direct evaluation of primary data.

The predefined inclusion and exclusion criteria were applied during the screening process.

Inclusion criteria:

- Original research articles published between 2000 and 2025;
- Studies investigating engineered drug delivery systems designed to modify the pharmacokinetic and/or pharmacodynamic properties of BE or BAs, such as nanoparticles, micelles, phytosomes, hydrogels, etc.;
- Exclusion criteria:
- Studies focusing exclusively on raw *Olibanum*, non-formulated extracts, or non-engineered formulation approaches aimed solely at improving solubility (e.g., physical blending with solubility enhancers, basic use of co-solvents or surfactants without defined carrier systems);
- Studies investigating multi-component herbal formulations where the specific contribution of *Boswellia*-derived constituents could not be clearly distinguished;
- Studies limited to the physicochemical characterization of formulations without in vivo or cellular evaluation of bioavailability or biological activity;
- Studies evaluating only formulated BE/BAs without a non-formulated control, which did not allow the assessment of the extent of improvement in bioavailability or biological activity attributable to the formulation;
- Studies focusing exclusively on topical delivery systems, as the scope of this review is limited to oral drug delivery systems;
- Essential oil-based formulations, as these do not represent BAs carrier systems;
- Studies on *Boswellia* species other than *Boswellia serrata*, to maintain taxonomic consistency.

**Figure 3:**
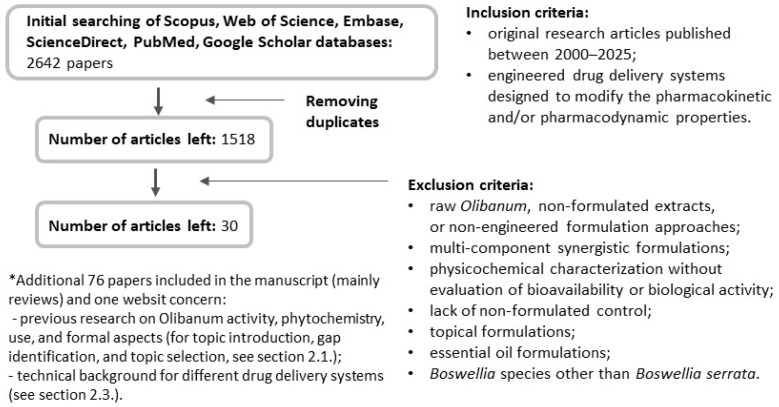
A flowchart of literature selection for analysis of *Boswellia* drug delivery systems.

Although the current review does not follow the formal framework of a systematic review, efforts were made to ensure comprehensive coverage of the available literature on novel *Boswellia* formulations. The study selection process is summarized in a flowchart (Figure 3).

Additionally, the patent literature was screened as part of the literature search strategy to identify potential formulation innovations for BE and BAs. However, most identified patents described non-engineered extract preparations or simple formulation approaches rather than structured drug delivery systems. Where patents reported drug delivery-related concepts, these were typically not supported by comparative experimental data against unformulated controls and, therefore, did not meet the predefined inclusion criteria of this review. As a result, patent documents were not included in the main analysis.

### 2.3. Use of Review Articles for Technical Background

Although review articles were not included as primary sources for data analysis or interpretation, selected reviews were consulted to provide a general background on specific formulation types (i.e., characteristics of delivery systems or carrier platforms). These sources were used descriptively to support the technical characterization of formulations and not for critical discussion of *Boswellia*-specific results.

## 3. Comparative Analysis of Conventional Boswellia Extracts/BAs vs. Modern Delivery Systems

Drug delivery systems, as advanced platforms designed to optimize the absorption, stability, and therapeutic performance of bioactive compounds, play a pivotal role in enhancing the clinical potential of natural products such as *Boswellia*-derived preparations. Table 1 provides a synthesized overview of the studies included in this publication to allow for a direct comparison of the key pharmacokinetic parameters and biological effectiveness across different BE/BAs formulations. In the subsequent sections, the corresponding formulations are characterized in detail, and their relevance to current advancements in *Boswellia*-focused drug delivery is critically discussed.

### 3.1. Lipid-Based Drug Delivery Systems (LBDDS)

**Figure 4:**
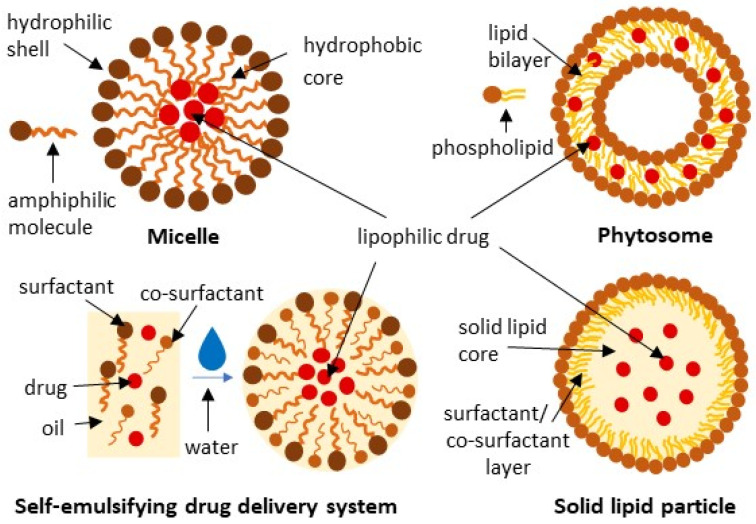
The types of lipid-based drug delivery systems tested for enhanced biological effectiveness of orally administered *Olibanum* extracts or boswellic acids (figure prepared by the author with the use of PowerPoint and Paint programs).

LBDDS are recognized as biocompatible carriers that enhance the delivery of poorly bioavailable drugs via oral, transdermal, pulmonary, or parenteral routes. They are formulated with vegetable or animal oils and additional surfactants and co-surfactants to provide benefits such as targeted drug delivery, reduced systemic toxicity, and/or protection against degradation, thereby improving the therapeutic effectiveness of the substances administered. LBDDS can be categorized into several types, including micellar formulations, vesicular systems, emulsions, and lipid particulate systems, each having unique properties and applications [80]. The various types of LBDDS that have been tested for their impact on the biological effectiveness of orally administered *Olibanum* extracts or BAs are illustrated in Figure 4 and discussed in the following section.

#### 3.1.1. Micellar Formulations

Micelles are colloidal nanostructures formed by amphiphilic molecules, with a hydrophobic core that can encapsulate lipophilic drugs and a hydrophilic shell that provides in vivo circulation stability (Figure 4). The shell can be further modified by adding ligands to provide active binding to target tissues and cells [81]. However, so far, the tested micellar formulations of BE have presented only moderate therapeutic benefits.

The single-dose crossover study of *Boswellia*-Loges^®^ micellar formulation and non-formulated BE (Biotikon^®^) demonstrated better pharmacokinetic properties for the micellar preparation, including increased C_max_ and AUC and decreased T_max_ (Table 2) [20]. Similar observations were made for the NovaSOL^®^ micellar formulation (10% of BE) tested in albino Wistar rats (n = 6) at a dose of 128 mg of total BAs/kg b.w. (body weight) in comparison to the corresponding dose of non-formulated extract. In this study, the C_max_ and AUC values increased, especially for AKBA (25-fold and 55-fold) and KBA (not detected after administration of native extract). However, the T_max_ values were similar for both preparations, which may be due to differences in methodology (animal and human tests) [50].

Despite divergences in the pharmacokinetic properties, no significant differences were observed between the *Boswellia*-Loges^®^ and Biotikon^®^ in terms of TNF-α (tumor necrosis factor alpha), IL-6 (interleukin 6), and IL-8 (interleukin 8) release from the lipopolysaccharide (LPS)-stimulated peripheral blood mononuclear cells (PBMCs) ex vivo. What is more, the decrease in the release of IL-1β (interleukin 1β) was higher for non-formulated BE [20]. Similarly, in another randomized study (the same conditions), despite a higher plasma concentration of BAs, the effects of both preparations on IFN-γ (interferon γ) and IL-10 (interleukin 10) levels in LPS-stimulated human whole blood cells ex vivo were the same. Moreover, in the T-cell receptor-activated lymphocytes in vitro, the decrease in the levels of IFN-γ, TNF-α, and IL-2, as well as the proliferation of lymphocytes, was higher for non-formulated BE [51]. These results suggested that preparations containing the non-formulated BE, however, with a sufficiently high content of BAs (about 10-times higher than in corresponding formulated BE), might be more effective in mitigating inflammatory disorders. Considering the possible explanation for the observed effects, it can be concluded that the problem was not the formulation itself, but the chemical used in its production. Polysorbate 20 is a surfactant derived from sorbitol and lauric acid, with polyethylene glycol (PEG) units. While it is considered to form lipid micelles (and not a polymer hybrid, where PEG is the underlying structural component; see Section 3.4.1), PEGylation may also influence the formulation properties to some extent. It is known that PEGylation is one of the strategies to improve drug stability by decreasing the drug’s recognition by inflammatory cells. Hence, targeting these cells with a PEG-containing formulation seems counterproductive [82]. On the other hand, the use of another substance as an emulsifier may have a completely different effect on inflammatory response, or this particular micellar formulation may have different molecular targets. All these issues should be addressed in future research. Finally, both the micellar formulation and the non-formulated BE did not affect PBMCs viability at 3–10 µg/mL [51]. However, a systematic toxicity assessment, including in vivo studies, is required to verify the safety of these formulations.

#### 3.1.2. Phytosomes (Vesicular System)

Phytosomes are formed when a stoichiometric amount of phospholipids reacts with plant extracts or compounds (1:1 or 2:1), forming a micellar-shaped structure in which the active principle is an integral part of the lipid bilayer through the hydrogen bonds (Figure 4). Phytosomes are readily transported from a hydrophilic environment into the lipid-friendly one of the enterocyte cell membrane, enhancing the intestinal absorption of substances that are poorly absorbed in their unchanged forms [83,84].

Indeed, in a randomized, crossover study with two treatments in 12 healthy people (fasting conditions), some pharmacokinetic parameters were significantly altered after oral administration of a phytosome *Boswellia* preparation, Casperome™, compared to non-formulated BE [19]. Casperome™ is a combination of a standardized BE and soy phosphatidyl choline (1:1), produced with Indena’s phytosome technology, with the addition of microcrystalline cellulose to improve stability. The administration of an equivalent dose of non-formulated BE and Casperome™ (which corresponds to approximately three-times lower BAs content in the Casperome™ group) resulted in about a 2-fold higher C_max_ of KBA, AKBA, AβBA, βBA, and αBA, as well as about 1.5-fold lower T_max_ (except KBA), meaning the significantly improved and quicker absorption in the Casperome™-treated group. Simultaneously, the T_1/2_ and K_e_ did not change (except for AKBA), suggesting that this formulation did not further affect the metabolism and elimination of BAs (Table 2).

There is also a report indicating the improved tissue distribution (in the brain, muscle, eye, liver, and kidney) of BAs after Casperome™ administration at a dose corresponding to 88 mg BAs/kg of body weight (in vivo animal studies, n = 6 Wistar rats/group), compared to the same dose of BAs in non-formulated BE [57]. Of particular importance was the marked increase in brain concentrations of KBA, AKBA, and βBA (35-fold, 12-fold, and 3-fold, respectively) in the Casperome™-receiving groups, which may lead to cognitive benefits [8]. However, the exact biological effects of Casperome™ in this regard still require clinical verification.

As of now, clinical studies of Casperome™ have examined its effects on irritable bowel syndrome, acute diarrhea, osteo-muscular pain, and asthma [52,53,54,55,56]. However, due to the lack of a non-formulated BE control, it cannot be determined whether its effectiveness is enhanced compared to non-formulated extracts. Nevertheless, considering the absorption and distribution studies of BAs from Casperome™, it is highly expected. To support this assumption, the phytosomal formulations of BE with Phospholipon^®^90H (the particle size 441 nm, entrapment efficiency 95–96%) and BA (12-ursene 2-diketone) with soy phosphatidyl choline (the particle size 508 nm) were tested and demonstrated to be more effective in relieving inflammation symptoms in the induced rat arthritis model (n = 6 or n = 3) than non-formulated BE or BA at an equivalent dose of 180 mg/kg b.w. or 100 mg/kg b.w., respectively. Depending on the study, the phytosomal formulations reduced paw thickness and paw volume, lowered TNF-α levels, and improved histopathological changes in bone tissue and cartilage [58,59]. Moreover, the zeta potential (−36.35 mV) has demonstrated colloidal stability. However, no long-term stability tests were conducted [58,59].

The last studied aspect of *Boswellia* phytosomal formulations is the addition of co-surfactants to the phospholipid-BE complex [85]. From different tested substances (vitamin E–TPGS, gelucire 44/14, pluronic f68, pluronic f127, Tween 80), the formulation composed of BE, phospholipid, and pluronic f127 (1:1:1) showed the most promising results, i.e., while the mass flux in the Caco-2 cell permeability model increased 8-fold (KBA) and 15-fold (AKBA) for basic phytosomes, for formulation with the co-surfactant, it was 27-fold and 42-fold higher compared to BE. Consequently, the plasma concentration of KBA and AKBA in the rat model (n = 3) increased, with C_max_ 26-fold and 14-fold higher for the novel formulation compared to BE. Unfortunately, this approach has not yet been further studied.

#### 3.1.3. Self-Emulsifying Drug Delivery Systems

Self-emulsifying drug delivery systems (SEDDS) are mixtures of drugs, oils, surfactants, and co-surfactants, which, in the aqueous environment of the gastrointestinal tract, spontaneously form microemulsions (SMEDDS) or nanoemulsions (SNEDDS), depending on the droplet size (Figure 4). They are easy to produce and effective at enhancing the solubility and absorption of poorly water-soluble drugs. However, the high concentrations of gut-irritating surfactants and the chemical instability of some drugs in SEDDS formulations limit their application [84,86]. A few drawbacks of using SEDDS can be addressed by employing, e.g., self-emulsifying hybrid particles, as described in Section 3.4.1.

As for the classical SEDDS, they were indeed shown to increase the oral bioavailability of BAs in the ICR mouse model (n = 4). For the SNEDDS of KBA and AKBA (“nanoVAILABLE BOSWELLIA”, 150–197 sizes and 0.13–0.17 polydispersity indexes depending on the pH) at 100 mg/kg b.w., administered as the suspension, the rose of the C_max_ and AUC were about 2-3-fold, with K_e_ and T_max_ not changed, compared to non-formulated BAs [60]. In another study, the pharmacological effect of the optimized SMEDDS formulation of BE (37.5% polysorbate 80, 12.5% PEG 400, 25% caprylic/capric triglycerides, 25% BE; size 495 nm, no precipitation after 24 h) was evaluated. A dose equivalent to 250 mg of BE/kg b.w. in hard gelatin capsules (BAs content of 66%) was used to treat the carrageenan-induced paw edema (n = 6 Wistar rats/group) [61]. As a result, the paw edema inhibition levels at one hour were 62.2 ± 3.26% for SMEDDS, 6.19% for non-formulated BE, and 73.6% for etoricoxib. Meanwhile, at five hours, the effectiveness of both BE and SMEDDS was about 50%. This showed that the main difference between SMEDDS and BE was the time of obtaining the maximal activity (5-fold lower for SMEDDS), suggesting faster absorption of BAs from SMEDDS, with approximately the same final effect. This may also imply either comparable C_max_ values or, more likely, a limited therapeutic effect despite a higher plasma concentration. This may be due to differences in the studied models and to the distinct chemicals used to prepare the tested formulations. For the first one, the composition is proprietary [60], while the second is known to contain polysorbate, PEG, and triglycerides [61]. As discussed in Section 4.2, the PEG may decrease the recognition of active substances by inflammatory cells. Thus, while the activity of SMEDDS after five hours was the same as for non-formulated BE, this does not rule out the differences in the circulatory contents of BAs.

#### 3.1.4. Solid Lipid Particles (Lipid Particulate System)

Solid lipid micro- or nanoparticles (SLP) are composed of a solid lipid core (triglycerides, fatty acids, steroids, etc.) surrounded by a surfactant/co-surfactant layer (phospholipids, pluronic f68, polysorbate 80, etc.), with a drug incorporated in the lipid core (Figure 4). They enhance drug stability and can be designed to provide controlled and sustained release, as well as to target specific tissues [87]. SLPs are also often the basis for creating hybrid formulations.

The commercial formulation of SLP (phosphatidyl choline complex, WokVida™, KBA 7%, AKBA 1%) decreased the permeability of KBA into human HHL-17 hepatocytes in vitro, resulting in about a 3-fold decrease in the concentration–time profile in comparison to non-formulated BE (WokVel™, KBA 2.3%, AKBA 0.3%). Therefore, the KBA hepatic clearance can be reduced, thereby prolonging its therapeutic effect. On the other hand, the AKBA concentration remained unchanged [62].

The favorable effect of the WokVida™ formulation on osteoarthritis of the knee was tested in a prospective, randomized, double-blind, double-dummy, placebo-controlled study (n = 18–22/group). Patients took 333 mg of SLP in capsules (equivalent to 100 mg of BE per capsule) or 333 mg of BE in tablets (and, accordingly, placebo capsules/tablets) three times a day after meals for 2 months. While the SLP seemed to exert better control over some proinflammatory cytokines (IL-2, IL-4, and IFN-γ), no significant differences in WOMAC (Western Ontario and McMaster Universities Osteoarthritis Index) and VAS (Visual Analog Scale) scores between the two groups were observed. However, treatment with SLP significantly reduced the need for rescue analgesics (100 mg of aceclofenac) compared with non-formulated BE (by 76% vs. 34% at the end of the study) [63]. This finding indeed indicates the better anti-arthritic properties, attributed to the enhanced oral bioavailability of BAs [64]. Noticeably, no adverse events were observed during a clinical evaluation that included complete blood count, liver function tests, renal function tests, urine analysis, and electrocardiogram [63].

### 3.2. Polymer-Based Drug Delivery Systems (PBDDS)

**Figure 5:**
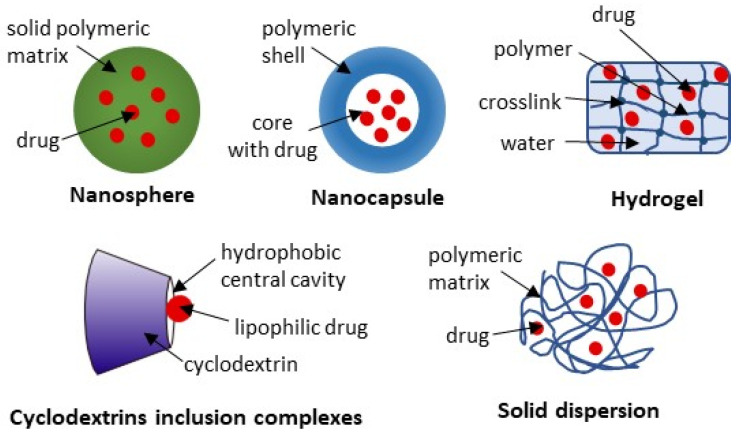
The types of polymer-based drug delivery systems tested for enhanced biological effectiveness of orally administered *Olibanum* extracts or boswellic acids (figure prepared by the author with the use of PowerPoint and Paint programs).

PBDDSs incorporate polymers of various origins for controlled and targeted drug release [88]. The polymers used may be natural (e.g., chitosan, cyclodextrin) or synthetic compounds, such as PLGA (poly(lactic-co-glycolic acid) and pluronic, which is poly(ethylene oxide)-poly(propylene oxide)-poly(ethylene oxide). PBDDS can be divided into many subtypes, including polymeric nanoparticles (nanospheres and nanocapsules), polymeric micelles, dendrimers, solid dispersions, polymer–drug inclusion complexes, polymer–drug conjugates, hydrogels, etc. [89]. Although polymer technology enables a precise adjustment of the preparation’s properties to meet specific needs (which is not always possible with lipid formulations), their complexity, advanced production techniques, and the need for strict control of production parameters to achieve reproducibility remain challenging [89,90]. Therefore, there are no commercial products based on the PBDDS for *Boswellia*, yet. However, some studies discussed in the following subsections gave promising results for different PBDDSs (Figure 5).

#### 3.2.1. Polymeric Nanoparticles (PNs)

PNs can be divided into nanocapsules with a core–shell structure and nanospheres with a solid matrix form (Figure 5). For the first one, a drug is typically located in the core and surrounded by the polymeric shell, whereas for the second, it is incorporated into the polymer matrix [89]. While drug release is driven by polymer degradation and/or drug diffusion through the polymer, the release kinetics are strictly determined by the structure of the carriers [91]. No less important are also the techniques for preparing the formulation or the proportions of the ingredients used, all of which give endless possibilities for controlled and targeted drug release [92]. The available studies of PNs designed for *Boswellia* delivery include chitosan and PLGA particles.

Chitosan is a biocompatible and biodegradable cationic polymer derived from chitin, readily soluble in dilute acidic solutions, with high mucoadhesive properties and the ability to be modified for improved solubility, drug targeting, etc. Chitosan-based PNs have been extensively tested, especially for cancer therapies, wound healing, and antibacterial treatments [93].

The BE-loaded chitosan nanospheres, prepared using the ionotopic gelation technique and optimized for a chitosan content of 0.2% dw and 1–1.5% of sodium tripolyphosphate (cross-linking agent), were tested in terms of cytotoxicity and antibacterial activity in vitro [65,66].

The used particles were 67.5 and 104.6 nm in size, with a polydispersity index of 0.127–0.147 (confirmed monodispersity), had an entrapment efficiency of 78–80%, favored conversion of crystalline BAs into the amorphous state during particle development, and were stated to be stable over 6 months (based on the zeta potential). The studies on the human lung cancer cell line A549 revealed enhanced apoptotic activity of BE-loaded nanoparticles compared to non-formulated BE, as evidenced by higher DNA fragmentation and the ability to arrest cells at the SubG_0_ phase of the cell cycle (22.75% cells vs. 5.26%). These effects were attributed to the increased cellular uptake of PNs [65]. In another study, these PNs were able to inhibit the growth of Gram-negative bacteria *Salmonella typhi* (MIC 3.91 µg/mL for PNs, 7.81 µg/mL for BE, and 0.5 µg/mL for positive control ciprofloxacin) [66], while BAs are typically credited with influencing Gram-positive bacteria [11]. On the other hand, no effect was observed for Gram-positive *Staphylococcus aureus*, which contradicts previous studies [11] and may be attributed to the specific properties of PNs. Nevertheless, we still cannot say what the effects of this formulation will be in vivo, or whether it will be delivered effectively to the site of a disease/infection.

Chitosan can also be modified to improve its physicochemical properties, e.g., solubility in aqueous media at both neutral and alkaline pH, whereas unmodified chitosan is soluble only at acidic pH [93,94]. Such a derivative, i.e., *O*-carboxymethyl chitosan, was used to produce AKBA-loaded nanospheres (size 132 nm, polydispersity index 0.112, entrapment efficiency 88%, and zeta potential 24 mV, i.e., benefit for crossing the blood–brain barrier). Their pharmacokinetic parameters, tissue distribution, and neuroprotective activity in a rat model of induced ischemic stroke (middle cerebral artery occlusion) were compared with those of pure AKBA at an equivalent dose of 10 mg/kg b.w. The plasma, brain, and liver concentrations at 2 and 6 h after administration increased by about 4-6-fold, 2-3-fold, and 1.5-fold, respectively, while the spleen and kidney concentrations decreased by about 1.5-fold. The higher mean residence time (MRT) and T_1/2_ (3-fold and 1.5-fold), and the lower K_e_ (3-fold), indicated a longer duration of the formulated drug in the body. The improved brain bioavailability resulted in decreased brain infarct volume and neurological deficit score. The enhanced release of antioxidant enzymes (SOD, superoxide dismutase; GSH-Px, glutation peroxidase) and diminished pro-inflammatory cytokine levels (TNF-α, IL-β) in the cortex through their effects on the Nrf2/HO-1 (nuclear factor erythroid 2–related factor 2/heme oxygenase-1) and NF-κB signaling pathways, as well as inhibition of 5-LOX, were shown to be responsible for the observed neuroprotection [67]. While in this study, the NPs were administered intravenously, which leaves the issues of oral absorption unverified, other papers indicated enhanced C_max_, T_max_, AUC, and T_1/2_ after oral intake of different drugs (including a boswellic acid) in the form of chitosan–NPs, and even higher values were observed for *O*-carboxymethyl chitosan derivative [10,93,94]. This observation was attributed to the high calcium-binding capacity of *O*-carboxymethyl chitosan, which disrupts cell junctions and enhances paracellular permeability of the epithelium, thereby increasing the absorption of PNs from the intestine [95].

PLGA, or poly(lactic-*co*-glycolic) acid, is one of the best-defined biomaterials available for drug delivery, with its properties influenced by a complex interplay of factors, such as the molecular weight, the ratio of lactic acid to glycolic acid, particle size, crystallinity, and the use of additional copolymers [96]. It offers not only high elasticity for PNs design but also introduces the need to consider these factors in detail to properly optimize the PLGA-based materials.

The pharmacokinetic and anti-inflammatory properties of the KBA and AKBA-loaded PLGA nanocapsules (the lactide/glycolide ratio 50:50) were tested in the carrageenan-induced paw edema model (n = 5 Sprague Dawley rats/group) at a dose of 50 mg of the drug per kg b.w. The PNs were prepared using the emulsion–diffusion–evaporation method and optimized based on their physicochemical properties. The final formulations, prepared with 1% polyvinyl alcohol as a surfactant and 10% trehalose as a cryoprotectant, had particle sizes of 153 and 180 nm, polydispersity indexes of 0.194 and 0.276, and entrapment efficiencies of 80–83%, and were stable at accelerated conditions for 3 months. The PLGA nanocapsules were produced by a sonification time of 2 min at 60% amplitude, with a polymer/drug ratio of 50:10. The observed values of C_max_, T_max_, AUC, and T_1/2_ increased for both KBA and AKBA nanocapsules, compared to non-formulated drugs, but to a slightly higher degree for AKBA (4.4-6-fold, 1.3-2-fold, 7-9-fold, 2-2.5-fold, respectively). As a consequence of higher oral absorption and longer duration of drugs in the body, the paw edema (paw volume) after five hours was inhibited by 61% and 67% for PLGA-KBA and PLGA-AKBA, in comparison to 35% and 40.5% for KBA and AKBA, and 43.5% for the control drug, ibuprofen, at the same dose [68,69].

#### 3.2.2. Hydrogels

Hydrogels are composed of a large amount of water (70–99%) and a crosslinked polymer network, which makes them solid-like structures (Figure 5). The high water content provides physical similarity to the body tissues, giving the hydrogels excellent biocompatibility [97]. They can be designed to offer targeted and controlled drug release through, e.g., pH-responsive swelling. For example, minimal swelling in the strongly acidic pH of the stomach can protect the drug from degradation or, conversely, protect gastric tissues from the irritating drug, which is, next, rapidly released and absorbed in the intestinal compartments. Moreover, the pH-triggered drug release is eagerly tested for cancer therapies, in which the environment is weakly acidic (pH 5.6–6.8), in contrast to normal tissues (pH about 7.4), offering the possibility of targeted cancer therapy [97,98].

This approach was also tested for BE (12.64% AKBA determined by isolation) in the induced colon tumorigenesis model in mice. The chitosan/sodium alginate/calcium chloride dual-crosslinked nanogel was prepared using ionic pregelation and polyelectrolyte complexation methods, yielding an encapsulation efficiency of 75%, a particle size of 105 nm, and a zeta potential indicating colloidal stability (−37.7 mV). The dose of 100 mg/kg b.w. per week of either BE or BE–nanogel, or 25 mg/kg b.w. of 5-fluorouracil, or the combination of BE-nanogel and 5-fluorouracil was administered intraperitoneally for 8 weeks. The pathological characteristics of colon lesions were scored, and the greatest reduction in aberrant crypt foci, i.e., morphological lesions representing an early stage of colon cancer, was observed for BE-nanogel and BE-nanogel with 5-fluorouracil. There was also a decrease in mitosis compared with placebo, but no differences between the tested treatments. On the other hand, the mRNA expression levels of proteins involved in tumor cell processes (Bcl2, B-cell lymphoma 2; MMP-9, matrix metalloproteinases 9; VEGF, vascular endothelial growth factor; COX-2, cyclooxygenase 2; and cyclin D1) were not changed in either group, emphasizing the need for further studies on the anti-cancer mechanisms of the BE-nanogel. Finally, to confirm the pH-sensitive behavior (for potential oral administration), the in vitro drug release was tested under simulated gastric and intestinal conditions. Indeed, the designed formulations were stable at pH 2.5 and released AKBA at pH 5 [70].

#### 3.2.3. Other PBDDS

Among other types of polymeric formulations tested for BAs delivery are inclusion complexes (IC) with cyclodextrins and solid dispersions [71]. Cyclodextrins are hydrophilic products of the enzymatic degradation of starch, with a hydrophobic central cavity that can accommodate lipophilic drugs, whereas in SD, the drug is dispersed throughout the water-soluble or easily hydrated matrix (Figure 5) [99,100]. Both formulations can enhance drug solubility in gastrointestinal fluids and, hence, improve oral absorption. Indeed, in an ex vivo intestinal absorption model using everted rat gut sacs, the absorption of the BE-loaded hydroxy propyl β-cyclodextrin complex (a more water-soluble cyclodextrin derivative) and pluronic f127-based solid dispersion of BE were about nine times and six times higher, compared to non-formulated BE [71]. However, how these observations can be translated into in vivo conditions, tissue distribution, and the biological effectiveness of BE has yet to be explained.

### 3.3. Inorganic Drug Delivery Systems (IDDS)

**Figure 6:**
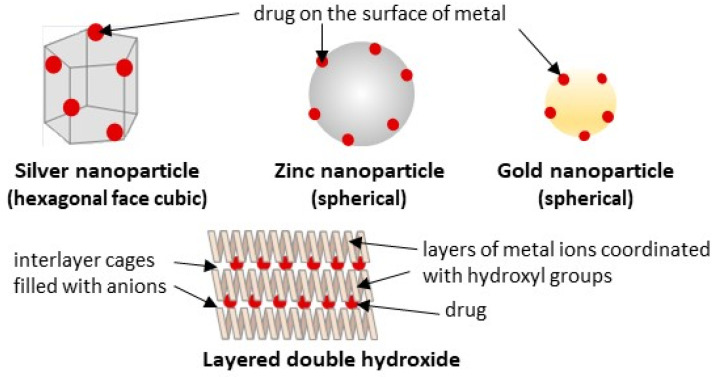
The types of inorganic drug delivery systems tested for enhanced biological effectiveness of orally administered *Olibanum* extracts or boswellic acids (figure prepared by the author with the use of PowerPoint and Paint programs).

IDDS can be categorized into metal-based and metal oxide-based formulations. They are highly stable and can offer tunable degradation rates and controlled drug release, but may exhibit unsatisfactory biocompatibility or biodegradability, especially with some metals, e.g., silver. However, these limitations may be minimized by modifying the IDDS structure and introducing hybrid formulations (see Section 3.4.2) [73]. The IDDS of the *Boswellia* products tested to date include silver, zinc, and gold nanoparticles, as well as lamellar solid layered double hydroxide (LDH) composed of magnesium and aluminum hydroxycarbonate (Figure 6).

#### 3.3.1. Metal-Based Nanoparticles

Metal-based nanocarriers are used less frequently as strictly drug delivery systems (unless additionally modified), but they benefit from the specific chemical and biological properties of different metals, e.g., the antibacterial and anti-inflammatory effects of silver. The possibility of additive/synergistic effects between nanocarriers and the loaded drug may increase the formulation’s therapeutic effectiveness, although this may not be due solely to enhanced drug bioavailability. Moreover, not only the type of metal but also the particle size and shape significantly affect interactions with drugs and cells, thereby influencing the absorption, activity, and toxicity of the final product [73,101].

The anti-inflammatory properties of hexagonal face cubic silver nanoparticles (AgNPs, size 277 nm, stability confirmed based on the spectral studies), coated with αBA and βBA, were tested in the carrageenan-induced rat paw edema model (n = 6 Wister albino rats/group). The doses of 500 and 2000 mg of BAs-loaded AgNPs per kg b.w., administered intraperitoneally, reduced paw edema to levels comparable to those of the standard control (healthy animals) and positive control (diclofenac 20 mg/kg b.w.), and this effect lasted from 30 min to 180 min. At the same time, the effect of non-formulated BAs was not significant [72]. Moreover, up to 2000 mg/kg, the nanoparticles were not toxic. However, this was an acute toxicity study (observation for 72 h), which does not exclude chronic toxicity, especially considering the reports about possible hepatic or kidney damage by AgNPs [101]. Finally, oral administration presents another unknown factor regarding their effective absorption and gastrointestinal toxicity [101]. While the polymer coating of AgNPs has previously been tested as an effective way to reduce silver toxicity, no such studies have been conducted for BAs.

Another metal-based formulation tested for anti-inflammatory activity was spherical zinc nanoparticles coated with a representative of BAs (the exact structure is not known, BAs-ZnNPs) [74]. The albino rats (n = 10/group) with induced ulcerative colitis received either 1 mg BAs-ZnNPs/kg b.w. or 500 mg BAs/kg b.w. five times per week orally for 6 weeks. As a result, BAs-ZnNPs significantly reduced levels of the immunoglobulins IgM and IgG, as well as the pro-inflammatory cytokines TNF-α, IL-1β, and IL-8, with a higher effectiveness compared to non-formulated BAs. The underlying mechanisms are consistent with the activity of BAs and include the downregulation of STAT-3 and PI3K protein levels and NF-κB and COX-2 gene expression. Moreover, in contrast to, e.g., silver, ZnNPs are considered less toxic and more biocompatible, since zinc is present in all body tissues as an important trace element for cellular signaling [73,74]. On the other hand, there were reports on some organ damage (e.g., liver, kidney, and spleen) after excessive exposure to ZnO-based NPs [73]. Hence, detailed toxicological studies should be conducted for this formulation. Moreover, potential stability concerns and unpredictable biological performance may arise, as the tested BAs-ZnNPs had a broad size range (50–100 nm, confirmed by a polydispersity index of 0.957), and were susceptible to agglomeration, with a zeta potential of −0.555 mV [74].

Finally, the βBA-coated spherical gold nanoparticles (βBA-AuNPs, sizes 13 or 24 nm, depending on the chemical or physical preparation method) were tested for their potential to inhibit the aggregation of tau protein, with a view to their prospective application in Alzheimer’s disease. The formulation indeed proved to be more effective than free βBA and unloaded AuNPs [76]. Similarly, in another study of βBA-AuNPs (size 27 or 55 nm), they were proven to effectively inhibit α-synuclein aggregation—a key pathological hallmark of Parkinson’s disease [75]. Importantly, in both studies, non-covalent βBA-GNPs showed the most significant effects, in comparison to covalently conjugated βBA. Although these were in vitro studies on isolated proteins, AuNPs were also shown to cross the blood–brain barrier (especially for particles < 20 nm) and to be highly stable and biocompatible [73].

#### 3.3.2. Layered Double Hydroxides (LDHs)

LDHs are a class of inorganic lamellar nanomaterials with a 2D structure consisting of positively charged brucite-like layers composed of metal ions coordinated with hydroxyl groups and interlayer cages filled with charge-balancing anions, where organic substances can be incorporated (Figure 6). They have low toxicity and high biocompatibility and can offer pH-dependent solubility, sustained drug release, and high membrane permeability, among other properties [102].

The LDH, composed of magnesium–aluminum hydroxide–carbonate loaded with BE (65% BAs), was tested for antimicrobial activity in vitro. In contrast to inactive free BE, BE-LDH reduced the viable colonies of Gram-positive and Gram-negative bacteria, *Escherichia coli* and *Staphylococcus aureus*, by 63% and 34%, respectively, while being inactive towards *Pseudomonas aeruginosa* and *Staphylococcus epidermidis*. Also, the BE-LDH was the only product to significantly inhibit the intracellular ROS generation in human bone marrow mesenchymal stem cells (hMSC) in vitro. Interestingly, calcinated LDH shows no antioxidant or antibacterial effects, despite a higher BAs-loading capacity (88% vs. 58%). During calcination, LDH can decompose into a mixed oxide, becoming more reactive toward organic anions, and then return to its original structure by absorbing anions and water from the environment. However, it turned out that the firm binding of BAs to calcinated LDH decreased their cellular bioavailability [77]. These findings suggest that LDH may be a better carrier for BAs. However, since LDHs are alkaline, they are highly degradable at an acidic pH in the stomach, which limits their oral administration [102]. Therefore, LDHs are often modified and combined with polymers to form hybrid formulations (see Section 3.4.2).

### 3.4. Hybrid Drug Delivery Systems (HDDS)

**Figure 7:**
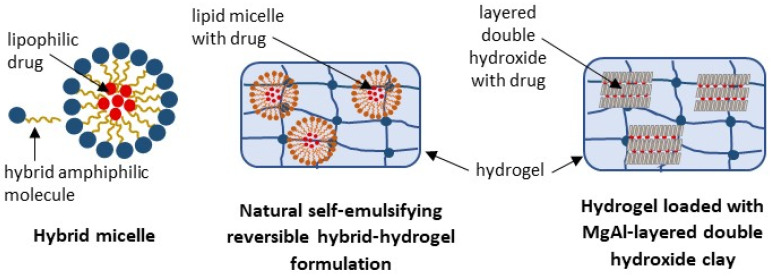
The types of hybrid drug delivery systems tested for enhanced biological effectiveness of orally administered *Olibanum* extracts or boswellic acids (figure prepared by the author with the use of PowerPoint and Paint programs).

To further improve drug delivery systems, hybrid formulations emerged. They can overcome the potential limitations of a single system by combining the properties of different particles. However, the features of the final product are not the simple sum of the characteristics of individual components, but rather a unique set of attributes resulting from complex interactions between the ingredients. The complexity of HDDSs makes predicting their behavior in biological systems difficult. Additionally, the intricate manufacturing processes make HDDS the most challenging delivery system [103,104,105]. To date, some polymer–lipid hybrid (PLH) and polymer–inorganic hybrid (PIH) drug delivery systems have been tested for BE, including the natural self-emulsifying reversible hybrid-hydrogel formulation, hybrid micelles, and a gellan gum-based hydrogel loaded with MgAl-layered double hydroxide clay (Figure 7).

#### 3.4.1. Polymer–Lipid Hybrid (PLH) Drug Delivery Systems

PLH drug delivery systems can provide the stability and controlled drug release characteristics of polymeric formulations, along with biocompatibility and high membrane retention, which are distinctive of lipid-based delivery systems [104,105]. They can be divided into self-emulsifying polymer–lipid hybrids, lipid-core polymer–shell or polymer-core lipid–shell structures, matrix polymer–lipid hybrids, and other variations [104]. Initial studies on the PLH of *Boswellia* include the natural self-emulsifying reversible hybrid–hydrogel formulation and hybrid micelles (Figure 7).

To prepare the natural self-emulsifying reversible hybrid–hydrogel formulation (N’SERH), the standardized BE (see Table 2) was emulsified with sunflower lecithin and then impregnated into the hydrogel matrix from fenugreek seed galactomannan by a gel-phase, thin-film dispersion process (FenuMat^®^ technology), followed by evaporation to the powder form. It resulted in the N’SERH system with an average particle size of 184 nm, which was stable for 6 months at accelerated conditions. In the gastrointestinal tract, this formulation can again swell into a hydrogel and self-emulsify, facilitating sustained release and absorption of active ingredients and maintaining their circulatory stability. Indeed, in the double-blinded, single-dose crossover study, the observed values of C_max_, T_max_, AUC, and T_1/2_ for all BAs increased following administration of N’SERH, compared to non-formulated BE. The observed increase was about 2-6-fold, despite an approximately 2-fold lower BAs concentration in the N’SERH (for details, see Table 2) [18]. Moreover, in the carrageenan-induced paw edema model (n = 5 Sprague–Dawley rats/group), the dose of 75 mg N’SERH/kg b.w. (about 15 mg BAs) reduced the edema after five hours by 70.5%, in contrast to 38% and 42% inhibition observed for the same dose of BE (corresponding to 35 mg BAs) and 10 mg/kg b.w. of diclofenac, respectively [18]. These results may be more favorable for the anti-inflammatory and anti-arthritic potential of the N’SERH formulation of BE, compared to the lipid and polymeric systems discussed above. However, direct comparisons across different studies are challenging. Nevertheless, *Boswellia* N’SERH appears to be a promising material for further in vivo testing.

Another type of formulation that may be classified as a hybrid one is dendrosomal encapsulation of βBA [79]. Despite the confusing nomenclature, these are not dendrimer-like forms, but micellar nanostructures with PEG-oleoyl ester as an amphiphilic molecule [106]. The higher impact on memory-related genes (CREB1, cyclic-AMP response element-binding protein 1; CREB2, cyclic-AMP response element-binding protein 2; BDNF, brain-derived neurotrophic factor; FMR1, fragile X messenger ribonucleoprotein 1; and MAP1B, microtubule-associated protein 1B) observed on the rat dopaminergic neuroblastoma B65 cell line was suggested to be due to the improved cellular uptake of the active compound [79]. However, the exact mechanism by which dendrosomal βBA enters cells remains to be investigated and confirmed in vivo. It may also be necessary to optimize the structure of dendrosomes and to modify them with ligands to target brain tissue [107]. Moreover, despite the improved impact on memory performance, this formulation reduced the viability of B65 cells, raising potential safety concerns.

#### 3.4.2. Polymer–Inorganic Hybrid (PIH) Drug Delivery Systems

PIH drug delivery systems combine the functionalities of both organic polymers and inorganic materials to create novel materials for controlled and targeted drug release and enhanced therapeutic efficacy. Their structures can be integrated, core–shell, or *Janus-like,* with the inorganic part being exposed on the surface or inside the particle [103]. In the case of BE, studies on PIH systems include a gellan gum-based hydrogel (GG) filled with MgAl-layered double hydroxide clay (LDH, previously loaded with 65% BAs) and cross-linked with Mg^2+^ (Figure 7).

The comparison of anti-inflammatory activity and effects on chondrogenesis for GG-BE and GG-LDH-BE was performed on hMSC in vitro. Their ability to inhibit the expression of pro-inflammatory genes, i.e., PGE2 (prostaglandin E2), COX-2, IL-1β, and TNF-α, was similar, and they enhanced the expression of SRY-Box Transcription Factor 9 (SOX9), a pivotal gene for chondrogenic differentiation, to a comparable level. Some differences were observed in the up-regulation of other chondrogenic genes. Specifically, ACAN (aggrecan) was more responsive to GG-LDH-BE activity, whereas COL2 (collagen type II) was more susceptible to GG-BE. Despite minor differences in activity, an important issue is their cytocompatibility (measured as hMSC metabolic activity), which was higher for GG-LDH-BE. Unfortunately, based on this study, we are unable to compare the results with those of non-formulated BE or LDH-BE alone [78]. Also, this was only a preliminary in vitro study, and so the in vivo bioavailability and effectiveness still require verification.

## 4. Challenges and Limitations in the Development of BAs Delivery Systems

### 4.1. Standardization of Boswellia serrata Extracts

The chemical profile of *Boswellia serrata* extracts varies widely depending on the resin source and extraction methodology, significantly affecting their pharmacokinetic and pharmacological properties [5]. Despite these variations, official guidelines, such as The European Pharmacopeia (Ph. Eur. 11.3, 2023) [15], often regulate only the quality requirements for the resin itself. Consequently, the specifications for the extracts can vary among producers, leading to inconsistent levels of BAs, particularly AKBA and KBA, in *Boswellia* preparations. In addition, commercial producers often standardize only the BAs content to a minimum declared amount, leading to uncertainties regarding their actual concentrations, as evidenced in numerous studies examining their biological effects. For example, the *Boswellia* extract used to produce Casperome™ (Phytosome) is claimed by the manufacturer to contain not less than 20% BAs, while independent verification found the actual content to be 70% [19]. Conversely, Biotikon^®^ is supposed to contain at least 85% BAs, but its verified level was only about 50%, well below the declared value [20]. However, in most studies, the content of BAs has not been verified. It was reported as merely what the manufacturer declared, or researchers used self-prepared extracts without any quality control [65,66].

The absence of harmonized quality standards and regulatory requirements for *Boswellia* extracts complicates cross-study comparison. For advanced delivery systems, the lack of standardized starting materials makes it difficult to optimize the formulation parameters, ensure batch consistency, and differentiate improvements attributable to delivery systems from those due to variations in extract quality.

### 4.2. Physicochemical and Stability-Related Limitations

Although novel delivery systems substantially enhance the solubility and pharmacokinetic features of BAs, they also introduce new physicochemical and stability-related challenges that can impede their translational potential. Consequently, stability considerations should be a fundamental aspect of developing and evaluating all BAs-based delivery systems. However, most published studies provide only short-term characterization, with long-term stability data available in just three studies [18]. In some cases, this may be due to confidentiality constraints, but still, it poses a significant obstacle to accurately comparing shelf life, batch-to-batch reproducibility, and the eventual commercial implementation of these systems. Importantly, these limitations pertain to physicochemical stability rather than structural transformation, as no evidence of covalent modification or alteration of the pentacyclic triterpene skeleton has been reported during the formulation processes.

Lipid-based delivery systems are particularly vulnerable to physicochemical degradation. The susceptibility of unsaturated lipids to peroxidation is a major source of instability and one reason for encapsulating them in hard or soft capsules [19]. However, encapsulation can bring its own set of challenges. For instance, hygroscopic excipients like PEG 400 can absorb moisture from gelatin capsule shells, leading to brittleness, deformation, or leakage. Higher concentrations of PEG 400 may additionally promote aggregate formation in aqueous environments [61]. Although optimization strategies generally aim to minimize PEG content, several studies suggest that PEG-containing systems may not be compatible with BE/BAs formulations at all [51]. The primary reason is that PEGylation stabilizes colloidal systems and extends their circulation time via steric shielding. However, this steric hindrance also reduces cellular uptake and diminishes the biological efficacy of BAs within immune cells, their main therapeutic targets [51]. Therefore, it is evident that excipient selection must balance storage and in vivo stability while considering the intended mechanism of action.

Polymer-based delivery systems are often regarded as more stable. However, their physicochemical behavior is highly dependent on the type of polymer and must align with the intended site of release. For example, chitosan, a weak polycation employed in BAs nanoparticles, is soluble and positively charged only at an acidic pH. Its protonated form facilitates mucoadhesion and epithelial permeation, but at neutral or alkaline pH, the polymer precipitates, loses its positive charge, and becomes structurally unstable, which limits its suitability for small-intestinal delivery [67]. In contrast, *O*-carboxymethyl chitosan remains water-soluble and anionic across a wide range of physiological pH, providing better colloidal stability and greater formulation usability in neutral or alkaline environments [95]. The differing physicochemical profiles of polymer-based systems directly influence BAs’ in vivo stability and release behavior.

Inorganic delivery systems present yet another set of challenges. For instance, layered double hydroxides (LDHs) tend to degrade significantly in acidic gastric conditions, which limits their oral applications [77]. Strategies such as polymer coating or polymer–inorganic hybridization can markedly enhance their stability [78], while calcination increases the loading efficiency of BAs (88% vs. 58%) but simultaneously decreases their cellular bioavailability due to the strong binding between LDH layers and anionic BAs [77]. These findings highlight the complex trade-offs between stability, loading efficiency, and biological performance of these systems.

Overall, despite notable advancements in formulation design, the systematic long-term stability of BAs delivery systems remains insufficiently researched. Lipid-based systems are prone to oxidation and physical transformations, polymeric carriers exhibit pH-dependent behavior or degradation, and some inorganic systems require structural modifications to withstand physiologically relevant environments. Additionally, limited data are available regarding the chemical stability of BAs within these systems under prolonged storage or stress conditions. While no evidence suggests structural transformation of the BAs core during formulation, their potential for degradation under specific physicochemical conditions (e.g., oxidative or pH stress) remains insufficiently investigated. Without thorough stability testing under both accelerated and real-time conditions, the true potential for translating BAs formulations into practical applications cannot be reliably evaluated.

### 4.3. Potential Toxicity and Safety Concerns

*Boswellia serrata* extracts are generally regarded as safe and have a long history of traditional use. However, the growing development of concentrated, BAs-enriched, and nano-formulated extracts introduces a range of toxicological uncertainties that go beyond those associated with conventional ones. Modern delivery technologies can fundamentally alter the pharmacokinetics and biodistribution of BAs, creating conditions where new risks may arise.

Advanced drug delivery systems significantly enhance the oral bioavailability of BAs, a crucial factor for therapeutic efficacy. However, this may also trigger off-target interactions, including cytotoxicity. It is essential to monitor organs that BAs previously had limited access to, such as the brain. Potential safety concerns arose, e.g., in studies of hybrid micelles, where, despite improved impact on memory performance, the formulation reduced the viability of the rat dopaminergic neuroblastoma cell line B65 at concentrations > 25 µM [79]. Additional risks are associated with the materials used in BE/BAs-containing nanocarriers. For instance, polymeric systems with PLGA may produce pro-inflammatory degradation products; inorganic carriers, including silver nanoparticles, can accumulate in organs, causing, e.g., hepatic or kidney damage; and SEDDS that employ high surfactant loads may irritate or disrupt gastrointestinal mucosa [101]. The safety of such systems relies not only on their chemical composition but also on particle size, surface charge, and degradation kinetics, which can vary between studies and complicate risk assessment.

Despite significant progress in formulation science, toxicological evaluation remains insufficient for most BE/BAs systems. The few available studies that assessed their potential in vivo risk focused on safety monitoring during the clinical trials of WokVida™ (solid lipid particles) and Casperome™ (phytosomes), as well as acute toxicity studies of silver nanoparticles in a rat model [72]. Still, high single doses used or short treatment durations do not reflect chronic supplementation patterns or long-term effects. Consequently, while traditional extracts are generally recognized as safe, the safety landscape for the next generation of BAs formulations remains incompletely defined and demands systematic, targeted investigation.

### 4.4. Interstudy Variability and Inconsistent Methodological Approaches

Research on BE/BAs delivery systems is characterized by substantial variability in experimental design, including differences in extract composition, doses, model selection (different cells, animals, or human studies), and analytical methods. Such heterogeneity limits cross-study comparability and complicates the assessment of the actual effectiveness for each delivery system. The frequent lack of direct comparisons to conventional extracts further complicates the problem. Consequently, several otherwise promising formulations have not been included in this review because inconsistent methodologies prevented their outcomes from being interpreted in a comparable framework. These discrepancies ultimately hinder the identification of truly effective delivery strategies and slow the progression of new formulations toward further development and translational evaluation.

### 4.5. Limited Clinical Evidence

Despite extensive preclinical research, there is a scarcity of clinical data confirming the superiority of advanced BE/BAs delivery systems. Additionally, the three human studies on the pharmacokinetic properties of BE formulations involved only a single administration and included a dramatically small number of participants (n = 8, 10, or 12). Moreover, these were healthy individuals, whereas it is known that drug metabolism can be affected by diseases, e.g., chronic inflammatory disorders, which are conditions treated with BAs. Such changes may include downregulation of transporters and drug-metabolizing enzymes, increased side effects, and complex drug–drug interactions (when taking other medications simultaneously), all of which can impact treatment outcomes [63]. Regarding the pharmacodynamic effects, only one study focusing on anti-arthritic properties of WokVida™ (solid lipid particles) was performed. However, the number of participants was also very low, i.e., 18–22 per group [63]. Clinical trials have also been conducted for Casperome™ (Phytosomes) [52,53,54,55,56]. However, due to the lack of a non-formulated BE control, they were not included in this review.

As a result, fundamental translational questions remain unresolved, including optimal dosing strategies, interindividual pharmacokinetic variability across diverse patient populations, and the clinical benefits of new formulations compared to conventional ones. Without well-designed clinical studies, the commercial adoption of BE-based advanced products is likely to remain limited.

## 5. Selection of BAs Delivery Systems: A Decision Framework Based on Therapeutic Objectives

**Figure 8:**
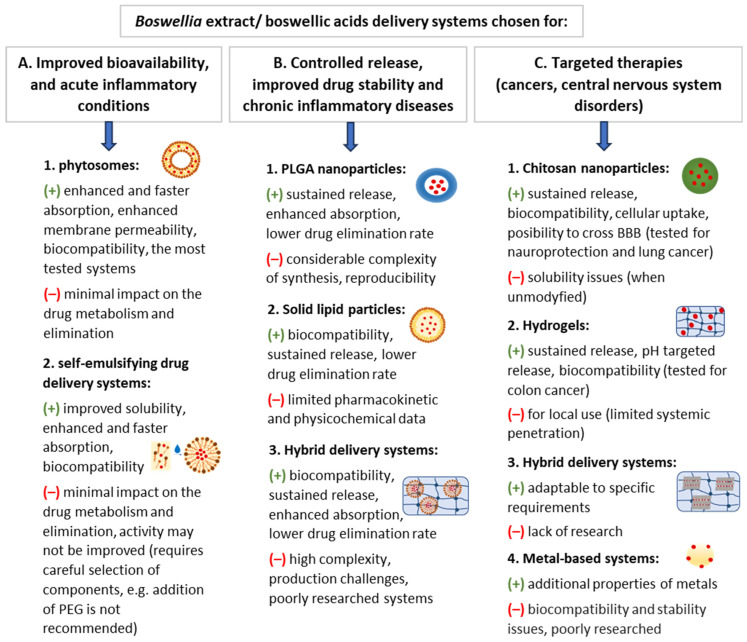
The decision flowchart for selection of *Boswellia* delivery system. BBB, blood–brain barrier; PLGA, (poly(lactic-co-glycolic acid); (+), advantages; (–) potential limitations. All icons were prepared by the author with the use of PowerPoint and Paint programs.

Choosing an effective delivery system for BAs requires addressing the differences in methodological approaches across studies and gaps in understanding how these systems work. Comparing studies is challenging due to the variations in the used extracts, doses, analytical methods, and in vivo models. Still, the current evidence allows us to build a framework for selecting the most promising formulations for different therapeutic goals. The flowchart in Figure 7 shows these selections based on the most consistently reported findings, highlighting the formulation features that are critical for each application. Overall, for acute inflammation, the best choice will be systems providing a rapid onset of action (increased C_max_ and AUC and decreased T_max_), i.e., lipid-based systems such as phytosomes or self-emulsifying systems, which are also the most tested and biocompatible formulations for these conditions. In contrast, chronic inflammatory diseases that require sustained exposure (e.g., osteoarthritis, inflammatory bowel disease) may align better with polymer-based systems, solid lipid particles, or lipid–polymer hybrids, which prolong residence time and support controlled release. Polymeric and hybrid nanocarriers are also preferable when tissue-specific release is needed. Inorganic systems, meanwhile, remain in the experimental stage due to potential toxicity and stability concerns. For more detailed information, refer to Figure 8 and previous chapters, where the advantages and disadvantages of various systems are discussed in depth. The intent of this model is not to prescribe a definitive formulation but to serve as a guide that aligns formulation properties with therapeutic needs, pharmacokinetic objectives, and translational potential. This decision-tree approach may serve as a foundation for future refinements as standardized methodologies and clinical data become available.

## 6. Future Perspectives

Despite advances in the delivery technologies of BAs, several challenges still hinder their broader translational progress. Breakthroughs will require more precise quality control of *Boswellia* extracts, nanocarrier engineering, and more exhaustive clinical evaluation.

Further development should start with well-defined and quality-controlled extracts. Establishing harmonized requirements for adjusting the contents of AKBA and other BAs within a narrow, defined range, aligned with the pharmacopeial definitions for quantified or standardized extracts, will help reduce variability and enable meaningful comparisons across studies.

Substantial opportunities lie in the continued development of intelligent, disease-responsive systems. Nanostructures decorated with targeting ligands or carriers engineered to respond to local biochemical stimuli, such as pH changes, oxidative stress, or enzymatic activity, could provide controlled drug release and preferential tissue accumulation. This might minimize systemic exposure while maximizing local concentrations at the site of pathology. Disease-specific strategies may also help expand the therapeutic scope of *Boswellia* products. Currently, most research focuses on osteoarthritis and general anti-inflammatory effects. However, BAs also show promise for treating neurological disorders, metabolic syndrome, asthma, and certain cancers. Developing delivery systems that can penetrate tissues previously inaccessible to BAs, such as those crossing the blood–brain barrier for neuroinflammation, could also unlock new applications for *Boswellia* extracts. Promising avenues include hybrid nanoplatforms that integrate lipid, polymer, and inorganic components. Finally, personalized and precision approaches based on patient phenotype or inflammatory biomarkers could optimize BAs therapy.

Future studies should focus on comparing the pharmacokinetics of advanced versus standard preparations in larger populations, conducting exposure–response analyses to identify effective plasma levels, exploring disease-specific mechanisms, and assessing long-term safety. Closer collaboration among laboratories, industry, and regulatory bodies is essential to define quality and efficacy standards for advanced BAs delivery systems.

## 7. Conclusions

Boswellic acids (BAs) are promising multi-target anti-inflammatory agents. However, their therapeutic performance is fundamentally hindered by poor aqueous solubility, low intestinal absorption, extensive metabolism, and overall low oral bioavailability. These challenges can be addressed with novel drug delivery systems that enhance oral absorption and systemic exposure to BAs, thereby improving their pharmacological outcomes compared with unformulated BAs.

This review provides a critical comparative assessment of conventional *Boswellia serrata* products and advanced delivery systems. It integrates evidence from pharmacokinetic studies and disease-specific models to identify the formulation strategies with the highest translational potential. The analysis also highlights critical gaps in quality control, long-term stability, safety assessment, and clinical verification of new *Boswellia* formulations. Despite these challenges, the review recommends rational formulation choices tailored to therapeutic use, emphasizing how the physicochemical characteristics of each system align with its intended clinical goals. For instance, while lipid-based phytosomes are, so far, the best-studied systems, their use should be limited to acute inflammatory conditions where a rapid onset of action is needed. In contrast, chronic inflammatory diseases, which require sustained exposure to BAs, would benefit more from polymer-based systems or lipid-polymer hybrids. Polymeric nanoparticles, hydrogels, and hybrid systems may offer advantages through tissue-specific release, potentially expanding the therapeutic applications of *Boswellia* products to conditions such as central nervous system disorders or cancers.

In summary, the evidence suggests that rationally engineered delivery systems can improve the biopharmaceutical profile and therapeutic potential of BAs. However, their full clinical value has yet to be established. Progress in this field will require collaborative efforts to standardize extracts, conduct rigorous long-term stability and safety tests, and execute systematically designed clinical trials. Focusing on these areas will transform BAs from inconsistent phytochemicals into clinically viable therapeutics with predictable efficacy and potential for regulatory approval.

## Figures and Tables

**Table 1:** A summary of the biological effects of different types of BE/BAs formulations.

	Type of Formulation	Plasma Profile of BAs (vs Non-Formulated Product), Tissue Distribution	Activity Summary (vs Non-Formulated Product), Potential Limitations	References
**Lipid-based drug delivery systems**	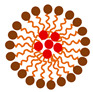 **Micellar formulations**	↑ C_max_, ↑ AUC, ↓ T_max_ (human studies; high-fat meal conditions); ↑ C_max_, ↑ AUC, not changed T_max_ (animal studies in rats)	anti-inflammatory properties not improved or ↓ (LPS-stimulated human PBMCs ex vivo; LPS-stimulated human whole blood cells ex vivo; T-cell receptor-activated lymphocytes in vitro); possibly due to PEGylation, which ↓ the recognition of the drug by inflammatory cells	[20,50,51]
	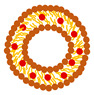 **Phytosomes**	↑ C_max_, ↑ AUC, ↓ T_max_, not changed K_e_ and T_1/2_ (human studies; fasting conditions); ↑ brain, muscle, eye, liver, and kidney concentrations (animal studies in rats)	↑ anti-arthritic properties (animal studies)—↓ paw thickness, paw volume, TNF-α level, improved histopathological changes in bone tissue and cartilage; clinical trials for the use in IBS, acute diarrhea, osteo-muscular pain, and asthma; however, without a non-formulated control	[19,52,53,54,55,56,57,58,59]
	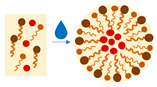 **Self-emulsifying drug delivery systems**	↑ C_max_, ↑ AUC, depending on the study not changed or ↓ T_max_, not changed K_e_ (animal studies in mice)	faster anti-inflammatory effect, but finally not improved (animal rat model)—paw edema inhibition at 1 h 62% vs. 6% for non-formulated extract, and at 5 h 50% for both formulated and non-formulated extract	[60,61]
	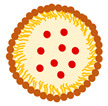 **Solid lipid particles**	↓ hepatic permeability (HHL-17 cells) for KBA—possibility for ↓ K_e_ and ↑ T_1/2_; no effect for AKBA; no in vivo data	↑ anti-arthritic properties—↓ levels of IL-2, IL-4, and IFN-γ, ↓ the need for rescue analgesics, no significant difference in WOMAC and VAS scores (human osteoarthritis); safe during the study (based on the blood count, liver function tests, renal function tests, urine analysis, and electrocardiogram)	[62,63,64]
**Polymer-based drug delivery systems**	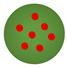 **Chitosan/CM-chitosan nanospheres**	↑ C_max_, ↑ AUC, ↑ T_max_, ↓ K_e_, ↑ T_1/2_ (animal studies); ↑ brain and liver, ↓ spleen and kidney concentrations (animal studies in rats)	↑ anticancer properties (lung cancer cells A549)—↑DNA fragmentation and a SubG_0_ phase arrest, ↑ antibacterial activity (*S. typhi*) (for chitosan); ↑neuroprotection (ischemic stroke, animal rat model)—↓ brain infarct volume and neurological deficit score, ↑ levels of SOD, GSH-Px, Nrf2, HO-1; ↓ levels of TNF-α, IL-β, NF-κB; ↓ activity of 5-LOX (for CM-chitosan)	[65,66,67]
	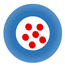 **PLGA nanocapsules**	↑ C_max_, ↑ AUC, ↑ T_max_, ↑ T_1/2_ (animal studies in rats)	↑ anti-inflammatory properties (paw edema, animal rat model)—↓ paw volume	[68,69]
	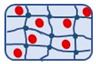 **Hydrogels**	no data	↑ anticancer properties of pH-sensitive nanogel (colon tumorigenesis, animal mouse model)—↓ aberrant crypt foci; expression levels of Bcl2, MMP-9, VEGF, and cyclin D1 not changed	[70]
	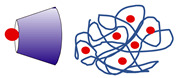 **Cyclodextrin inclusion complexes or solid dispersions**	no in vivo data; ↑intestinal absorption (ex vivo studies on everted rat gut sacs)	no data	[71]
**Inorganic drug delivery systems**	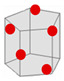 **Silver nanoparticles**	no data	↑ anti-inflammatory properties (paw edema, animal rat model)—↓ paw volume (however, tested only intraperitoneally); not toxic during acute toxicity studies (however, the possibility of chronic or gastric toxicity not excluded)	[72]
	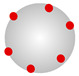 **Zinc nanoparticles**	no data	↑ anti-inflammatory properties (ulcerative colitis, animal rat model)—↓ levels of IgM, IgG, TNF-α, IL-1β, IL-8, STAT-3, PI3K, and NF-κB and COX-2 expression; potentially less toxic than Ag nanoparticles, but toxicity not verified; stability concerns	[73,74]
	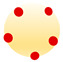 **Gold nanoparticles**	no data	↓ aggregation of tau protein and α-synuclein (in vitro)—the possible use in Alzheimer’s and Parkinson’s diseases (↑ activity for non-covalent particles); possibility to cross blood–brain barrier	[75,76]
	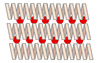 **Layered double hydroxides**	no data	↑ antibacterial activity (*E. coli* and *S. aureus*); ↑ antioxidant potential (hMSC cells in vitro) –↓ of intracellular ROS generation; potential stability challenges in acidic pH in the stomach	[77]
**Hybrid drug delivery systems**	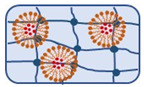 **Natural self-emulsifying reversible hybrid-hydrogel formulation**	↑ C_max_, ↑ AUC, ↑ T_max_, ↑ T_1/2_ (human studies; fasting conditions)	↑anti-inflammatory properties (animal rat model)— ↓ paw edema	[18]
	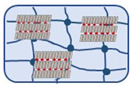 **Gellan gum-based hydrogel loaded with MgAl-layered double hydroxide clay**	no data	anti-inflammatory properties and chondrogenic gene expression similar to hydrogel formulation (hMSC cells); however, without a non-formulated control; ↑cytocompatibility (hMSC metabolic activity)	[78]
	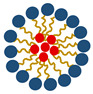 **Hybrid micelles**	no data	↑ impact on memory related genes (rat neuroblastoma B65 cells)—↑ levels of CREB1, BDNF, and FMR1 at 24 h and 72 h; ↓ or ↑ levels of CREB2 depending on concentration; and ↓ levels of MAP1B; ↑ toxic effect (↓B65 cells viability)	[79]

Abbreviations: 5-LOX, 5-lipoxygenase; AKBA, acetyl-11-keto-β-boswellic acid; AUC, area under the concentration vs time curve from time 0 to the last measured concentration; B65, neuronal cells line; BAs, boswellic acids; Bcl2, B-cell lymphoma 2; BDNF, brain-derived neurotrophic factor; BE, *Boswellia* extract; C_max_, maximal plasma concentration; CM-chitosan, *O*-carboxymethyl chitosan; COX-2, cyclooxygenase 2; CREB1, cyclic-AMP response element-binding protein 1; CREB2, cyclic-AMP response element-binding protein 2; FMR1, fragile X messenger ribonucleoprotein 1; GSH-Px, glutation peroxidase; HHL-17, an immortalized hepatocyte cell line; hMSC, human mesenchymal stem cells; HO-1, heme oxygenase-1; IBS, irritable bowel syndrome; IFN-γ, interferon γ; IgG, immunoglobulin G; IgM, immunoglobulin M; IL-1β, interleukin 1β; IL-2, IL-4, IL-8, interleukins 2, 4, 8; KBA, 11-keto-β-boswellic acid; K_e_, elimination rate constant from the central compartment; LPS, lipopolysaccharide; MAP1B, microtubule-associated protein 1B; MMP-9, matrix metalloproteinases 9; NF-κB, nuclear factor kappa-light-chain-enhancer of activated B cells; Nrf2, nuclear factor erythroid 2–related factor 2; PBMCs, peripheral blood mononuclear cells; PI3K, phosphatidylinositol 3-kinase; SOD, superoxide dismutase; STAT-3, signal transducer and activator of transcription 3; T_1/2_, time taken for the plasma concentration to fall to half of its original value; T_max_, time required to C_max_; TNF-α, tumor necrosis factor alpha; VAS, Visual Analog Scale; VEGF, vascular endothelial growth factor; WOMAC, Western Ontario and McMaster Universities Osteoarthritis Index; ↑, increase/higher; ↓, decrease/lower. All icons were prepared by the author with the use of PowerPoint and Paint programs.

**Table 2:** Plasma profile of BAs after administration of non-formulated and corresponding formulated *Boswellia* extracts based on human studies.

Tested Parameter	Study Design/Subjects												Ref.
	Non-Formulated Extract						Formulated Extract						
	Human studies; open-label, single-dose crossover study (n = 10/group, health subjects); high-fat meal conditions.												[20]
	Biotikon^®^ (BAs min. 85%); dose 800 mg extract p.o. in hydroxy-propyl-methyl-cellulose capsules; total six BAs 393.92 mg (KBA 57.12 mg, AKBA 20.16 mg, βBA 193.04 mg, *α*BA 66.32 mg, AβBA 19.52 mg, A*α*BA 18 mg)						*Boswellia*-Loges^®^ (micellar formulation, polysorbate 20 as emulsifier, glycerine as humectant); dose 800 mg p.o. in gelatine capsules; total six BAs 39.92 mg (KBA 5.44 mg, AKBA 3.44 mg, βBA 17.36 mg, *α*BA 6 mg, AβBA 3.04 mg, A*α*BA 2.88 mg)						
	KBA	AKBA	βBA	*α*BA	AβBA	A*α*BA	KBA	AKBA	βBA	*α*BA	AβBA	A*α*BA	
C_max_ (nM)	527	69	1491	656	1080	394	↑ 1182 *	↑ 317 *	↑ 2847 *	↑ 1236 *	↑ 2296 *	↑ 1080 *	
T_max_ (h)	5.0	5.0	6.0	6.5	7.0	7.0	↓ 2.1 *	↓ 1.5 *	↓ 4.5 *	↓ 4.5 *	↓ 4.5 *	↓ 5.0 *	
AUC (nM*h)	2684	218	29,963	12,378	13,087	4767	↑ 6772 *	↑ 1326 *	↑ 58,073 *	↑ 27,487 *	↑ 39,353 *	↑ 18,829 *	
T_1/2_ (h)	2.1	2.0	16.0	15.2	13.4	15.8	const.	const.	const.	const.	↓ 10.3 *	↓ 11.0*	
K_e_ (h^−1^)	0.325	0.340	0.043	0.046	0.052	0.044	const.	const.	const.	const.	↑ 0.068 *	↑ 0.063*	
	Human studies; open-label, randomized, crossover study with two treatments (n = 12, health subjects); fasting conditions.												[19]
	*Boswellia* extract (BE, triterpenes min. 25%, BAs min. 20%); dose 500 mg p.o. in hard gelatin capsules; total six BAs 353.88 mg (KBA 32.88 mg, AKBA 24.8 mg, βBA 94.56 mg, *α*BA 41.42 mg, AβBA 80.02 mg, A*α*BA 80.20 mg)						Casperome™ (Phytosome), BE + soy phosphatidyl choline (1:1) + microcrystalline cellulose; dose 500 mg p.o. in hard gelatin capsules; total six BAs 134.52 mg/capsule (KBA 11.62 mg, AKBA 8.22 mg, βBA 41.26 mg, *α*BA 18.38 mg, AβBA 27.52 mg, A*α*BA 27.52 mg)						
	KBA	AKBA	βBA	*α*BA	AβBA	A*α*BA	KBA	AKBA	βBA	*α*BA	AβBA	A*α*BA	
C_max_ (nM)	151	12	383	132	201	241	↑ 255 *	↑ 28 *	↑ 742 *	↑ 263 *	↑ 348 *	↓ 134 *	
T_max_ (h)	3.3	2.5	6.3	5.9	5.9	6.9	const.	↓ 1.3 *	↓ 4.1 *	↓ 4.0 *	↓ 4.3 *	↓ 5.0 *	
AUC (nM*h)	2083	38	8073	2998	3453	4056	const.	↑ 53 *	↑ 11,484 *	↑ 4263 *	↑ 4944 *	↓ 2411 *	
T_1/2_ (h)	15.4	5.51	26.12	31.76	23.57	23.55	const.	↓ 1.8 *	const.	const.	const.	const.	
K_e_ (h^−1^)	0.08	0.15	0.03	0.02	0.03	0.03	const.	const.	const.	const.	const.	const.	
	Human studies; randomized double-blinded, single-dose crossover study (n = 8/group health subjects); fasting conditions.												[18]
	*Boswellia* extract (BE); dose 400 mg p.o. in microcrystalline cellulose and maltodextrin capsules (2:1); total six BAs 187.88 mg (KBA 15.56 mg, AKBA 42.4 mg, βBA 69.52 mg, *α*BA 21.96 mg, AβBA 28.76 mg, A*α*BA 9.68 mg), *α*-thujene 0.2 mg						Natural self-emulsifying reversible hybrid hydrogel (N’SERH), i.e., BE emulsion into the galactomannan hydrogel matrix; dose 400 mg p.o. in microcrystalline cellulose and maltodextrin capsules (2:1); total six BAs 80.44 mg (KBA 4.0 mg, AKBA 42.0 mg, βBA 18.4 mg, *α*BA 6.0 mg, AβBA 7.6 mg, A*α*BA 2.44 mg), *α*-thujene 12.16 mg						
	KBA	AKBA	βBA	*α*BA	AβBA	A*α*BA	KBA	AKBA	βBA	*α*BA	AβBA	A*α*BA	
C_max_ (nM)	199	31	184	80	110	35	↑ 584	↑ 119	↑ 504	↑ 208	↑ 176	↑ 112	
T_max_ (h)	2.2	1.9	4.7	4.3	4.3	4.3	↑ 6.5	↑ 4.3	const.	↑ 6.8	↑ 7.1	↑ 6.5	
AUC (nM*h)	1899	97	1363	885	574	219	↑ 7624	↑ 583	↑ 5951	↑ 2527	↑ 2340	↑ 1119	
T_1/2_ (h)	8.5	3.9	7.2	12.0	9.9	6.3	↑ 16.2	↑ 5.7	↑ 9.2	↑ 16.5	↑ 17.4	↑ 10.1	

AKBA, acetyl-11-keto-β-boswellic acid; AUC, area under the concentration vs. time curve from time 0 to the last measured concentration; AαBA, acetyl-α-boswellic acid; AβBA, acetyl-β-boswellic acid; BAs, boswellic acids; BE, *Boswellia* extract; C_max_, maximal plasma concentration; KBA, 11-keto-β-boswellic acid; K_e_, elimination rate constant from the central compartment; T_1/2_, time taken for the plasma concentration to fall to half of its original value; T_max_, time required to C_max_; αBA, α-boswellic acid; βBA, β-boswellic acid; p.o., per os; Ref., references; ↑, increase; ↓, decrease; const., not changed; * statistical significance (*p* < 0.05) according to the source article.

## Data Availability

No new data were created or analyzed in this study. Data sharing is not applicable to this article.

## Abbreviations

The following abbreviations are used in this manuscript:

5-LOX	5-lipoxygenase
ACAN	aggrecan
AgNPs	silver nanoparticles
AKBA	acetyl-11-keto-β-boswellic acid
AUC	area under the concentration vs. time curve from time 0 to the last measured concentration
AuNPs	gold nanoparticles
AαBA	acetyl-α-boswellic acid
AβBA	acetyl-β-boswellic acid
BAs	boswellic acids
BBB	Blood–brain barrier
Bcl2	B-cell lymphoma 2
BDNF	brain-derived neurotrophic factor
BE	*Boswellia* extract
b.w.	body weight
C_max_	maximal plasma concentration
COL2	collagen type II
COX-2	cyclooxygenase 2
CREB1	cyclic-AMP response element-binding protein 1
CREB2	cyclic-AMP response element-binding protein 2
dw	dry weight
FMR1	fragile X messenger ribonucleoprotein 1
GG	gellan gum-based hydrogel
GSH-Px	glutation peroxidase
HDDS	hybrid drug delivery systems
hMSC	human bone marrow mesenchymal stem cells
IC	inclusion complexes
IDDS	inorganic drug delivery systems
IFN-γ	interferon γ
IL-10	interleukin 10
IL-1β	interleukin 1β
IL-2	interleukin 2
IL-4	interleukin 4
IL-6	interleukin 6
IL-8	interleukin 8
JAK/STAT	Janus kinase/signal transducer and activator of transcription
KBA	11-keto-β-boswellic acid
K_e_	elimination rate constant from the central compartment
LBDDS	lipid-based drug delivery systems
LDH	lamellar solid layered double hydroxide
LPS	lipopolysaccharide
MAP1B	microtubule-associated protein 1B
MAPKs	mitogen-activated protein kinases
MMP-9	matrix metalloproteinases 9
MRT	mean residence time
N’SERH	natural self-emulsifying reversible hybrid-hydrogel formulation
NF-κB	nuclear factor kappa-light-chain-enhancer of activated B cells
Nrf2	nuclear factor erythroid 2–related factor 2
Nrf2/HO-1	nuclear factor erythroid 2–related factor 2/heme oxygenase-1
PBDDS	polymer-based drug delivery systems
PBMCs	peripheral blood mononuclear cells
PEG	polyethylene glycol
PGE2	prostaglandin E2
PI3K/Akt	phosphatidylinositol 3-kinase/protein kinase B
PIH	polymer-inorganic hybrid
PLGA	poly(lactic-co-glycolic acid)
PLH	polymer–lipid hybrid
PNs	polymeric nanoparticles
SEDDS	self-emulsifying drug delivery systems
SLP	solid lipid particles
SNEDDS	self-nano-emulsifying drug delivery systems
SOD	superoxide dismutase
SOX9	SRY-Box transcription factor 9
T_1/2_	time taken for the plasma concentration to fall to half of its original value
TLR4	toll-like receptor 4
T_max_	time required to C_max_
TNF-α	tumor necrosis factor alpha
VAS	visual analog scale
VEGF	vascular endothelial growth factor
WOMAC	Western Ontario and McMaster Universities Osteoarthritis Index
ZnNPs,	zinc nanoparticles
αBA	α-boswellic acid
βBA	β-boswellic acid
